# The Pre-Implementation Phase of a Project Seeking to Deliver a Community-Based CVD Prevention Intervention (SPICES-Sussex): A Qualitative Study Exploring Views and Experience Relating to Intervention Development

**DOI:** 10.1177/15248399231182139

**Published:** 2023-06-30

**Authors:** Thomas Grice-Jackson, Imogen Rogers, Elizabeth Ford, Harm Van Marwijk, Catherine Topham, Geofrey Musinguzi, Hilde Bastiaens, Linda Gibson, Mark Bower, Papreen Nahar

**Affiliations:** 1 Brighton and Sussex Medical School, Brighton, UK; 2 National Institute for Health Research, London, UK; 3 Makerere University, Kampala, Uganda; 4 Universiteit Antwerpen, Antwerpen, Belgium; 5 Nottingham Trent University, Nottingham, UK

**Keywords:** Community Intervention, Community Organization, Health Research, Health Promotion, Cardiovascular disease, Health Education, Community-Based Participatory Research, Qualitative Research, Community Assessment, Health Disparities

## Abstract

**Background.:**

Community-led health care interventions may be an effective way to tackle cardiovascular disease (CVD) risk factors, especially in materially deprived communities where health care resources are stretched and engagement with institutions is often low. To do so effectively and equitably, interventions might be developed alongside community members through community engagement.

**Objectives.:**

The aim of this project was to carry out stakeholder mapping and partnership identification and to understand the views, needs, experiences of community members who would be involved in later stages of a community-based CVD prevention intervention’s development and implementation.

**Methods.:**

Stakeholder mapping was carried out to identify research participants in three communities in Sussex, United Kingdom. A qualitative descriptive approach was taken during the analysis of focus groups and interviews with 47 participants.

**Findings.:**

Three themes were highlighted related to intervention design (a) Management: the suitability of the intervention for the community, management of volunteers, and communication; (b) Logistics: the structure and design of the intervention; and (c) Sociocultural issues, the social and cultural expectations/experiences of participants and implementers.

**Conclusions.:**

Study participants were open and willing to engage in the planned community-based intervention, particularly in elements of co-design and community-led delivery. They also highlighted the importance of sociocultural factors. Based on the findings, we developed recommendations for intervention design which included (but were not limited to): (a) a focus on a bottom-up approach to intervention design, (b) the recruitment of skilled local volunteers, and (c) the importance of fun and simplicity.

In high-income countries, cardiovascular diseases (CVD) remain the largest cause of mortality despite improving trends. The World Health Organization (WHO) estimates that 80% of worldwide CVD fatalities are preventable (World Health Organization, 2018) and the U.K. Biobank Prospective Study shows that behavior changes such as improving dietary patterns (Gao et al., 2021; Petermann-Rocha et al., 2021) or increasing exercise (Laukkanen et al., 2020) can significantly improve CVD morbidity and mortality. Furthermore, socio-economic deprivation acts as a risk factor for CVD and significantly predicts CVD morbidity and mortality (Kimenai et al., 2022).

In 2009, the U.K. Department of Health and the National Health Service (NHS) launched the Health Check program through the CVD prevention strategy (NHS, 2009). This includes a risk assessment of 40- to 70-year-olds without known CVD-related conditions, followed by a diagnostic referral for those assessed as high-risk, and behavioral interventions and signposting for those at medium risk of developing CVD. Modeling predicted that Health Checks would be cost-effective and could prevent 2,000 deaths and 9,500 CVD events annually with universal uptake of the program (NHS, 2008); however, uptake has been variable with poor follow-up referral rates (Robson et al., 2016). The program has also performed poorly in reaching economically disadvantaged communities and does little to address health inequalities related to socioeconomic status (Visram et al., 2015). Rather than adopting this universal and top-down approach to CVD health provision, care-providers could consider looking to local communities to assist in the development and delivery of care.

In low- and middle-income countries (LMIC), there is evidence for the successful implementation of community-based interventions that increase knowledge of, and change behavior related to, CVD (Hassen et al., 2022). However, their use in the Global North is less well tested or understood (Hassen et al., 2021). These community-based strategies often make use of task-sharing approaches whereby non-professional or non-specialist community members are trained and facilitated to provide basic care in local communities (Anand et al., 2018). These approaches are particularly useful in resource limited or unstaffed health care systems and for health care issues where the complexity of care is low, but levels of person-to-person interaction are high, such as public health and CVD care (Gaziano et al., 2015; Ndejjo et al., 2020). While primarily utilized in LMICs, community-based approaches which make use of community health workers (CHWs) are being increasingly adopted in high-income countries where health care systems struggle to meet the needs of patients with chronic health conditions which can be ameliorated by behavior changes (Aerts et al., 2022; Le Goff et al., 2021; Perry et al., 2014). CHW can be effective in high-income counties because of their effective use of social networks, social capital and mutual trust among marginalized communities (Saint Onge & Brooks, 2021) and because of their cost effectiveness (Jacob et al., 2019).

Projects and interventions that make use of CHW can also adopt community-based participatory research (CBPR) practices and community engagement (CE) principles and practice to improve care. CBPR literature emphasizes the equitable involvement of both community and academic partners throughout the research process (Brush et al., 2020). CE is the process of working collaboratively with and through groups of people affiliated by geography to address issues affecting the well-being of those people” (Brogan & McCloskey, 2021).

Literature on CBPR and CE emphasize a push for human-centered research design (Van Velsen et al., 2015). Yardley (Yardley et al., 2015) focused on this idea in their “person-based” approach to digital health interventions, where they recommended “understanding and accommodating the perspectives of the people who will use the intervention.” Hopkins and Rippon’s (Hopkins & Rippon, 2015) “asset-based” approach to CE interventions recommends recognizing and adapting to the needs, wants and strengths already present in the community. Such an implementation approach requires flexibility and adaptability, as well as deep involvement with the community. Furthermore, CE and CBPR recommend that participants and community stakeholders should be involved at every level of project planning through co-design. Yardley et al. included this as a key element of their paper, writing that people from the priority population should be involved in project development as well as at every stage of the implementation (Yardley et al., 2015). These processes require high levels of trust and participation from the community, which has its own challenges. Trust especially takes significant time and resources to develop and is an under-studied area of community engagement (Lucero et al., 2020; Moore et al., 2015).

The SPICES project (Scaling-up Packages of Interventions for Cardiovascular disease prevention in selected sites in Europe and Sub-Saharan Africa) is an international collaboration between countries in the Global North (Sussex and Nottingham, United Kingdom; Brest, France; Antwerp, Belgium) and Global South (Kampala, Uganda; Limpopo, South Africa) which aims to develop and implement community-based behavioral interventions to reduce CVD risk factors. The United Kingdom’s Sussex site, “SPICES-Sussex,” aimed to work with community members to co-design and implement a community-based CVD risk reduction intervention in several communities in Sussex. The project was split into three phases (Nahar et al., 2020) which included

1. Pre-implementation: This involved an assessment of community views and needs, stakeholder mapping, and design and development of the initial intervention parameters.
2. Per-implementation phase: This involved co-design of intervention parameters, recruitment and training of community health workers to deliver the intervention, implementation of the intervention, and mixed-method data collection.
3. Post implementation phase: This included data analysis and evaluation of key outcome measures, a mixed process evaluation using the RE-AIM framework.

This paper presents the process and results of the formative evaluation conducted during the pre-implementation phase. The evaluation was designed to achieve the following objectives:

1. Conduct stakeholder mapping to identify key partner organizations through which the community health volunteer led intervention could be delivered,
2. Evaluate community and volunteer views and perspectives on the planned intervention,
3. Identify key recommendations for design and delivery of the intervention

These objectives laid the groundwork for the per-implementation phase, which was designed to center stakeholder and community perspectives in the co-design process, including selection of community partnerships. Beyond Sussex and the SPICES project, we offer recommendations for project managers developing and implementing community-based health interventions more broadly.

## Methods

### Study Design

This study and the wider SPICES project are taking a Community-Based Participatory Research approach to intervention design and development alongside a realist approach to the evaluation meaning that the research and any intervention developed will be developed for the local community context (Abma et al., 2019; Jagosh et al., 2012, 2015; Pawson, 1997). Rather than focusing on representativeness and Generalizability the research seeks to identify the mechanisms that can be used to achieve positive outcomes in the specific community.

A qualitative descriptive approach was taken in this study. A series of focus groups and interviews were carried out with stakeholders identified during the stakeholder mapping phase of the research. The Consolidated criteria for REporting Qualitative research (COREQ) checklist has been completed for the reporting of this manuscript (Tong, 2007). The study received ethical approval from BSMS’s Research Governance and Ethics Committee (RGEC) (ER/BSMS9JPY/3).

### Stakeholder Identification, Sampling and Recruitment

Marginalized communities in the local area, with lower socio-economic status (SES), were identified for the study population using the Indices of Multiple Deprivation (Kontopantelis et al., 2018; Ministry of Housing, Communities & Local Government UK Government, 2019). Three locations within the county of Sussex were selected which had IMD scores of 3 or less (Kontopantelis et al., 2018). They included (a) East Brighton, (b) Newhaven, and (c) Hastings and Rother.

To effectively engage with the community, it is important to identify and map key stakeholders who can serve as gatekeepers of the community. Such mapping enables researchers to determine the extent to which stakeholder missions, visions, and activities align with the study objectives (BSR, 2011; Nahar et al., 2020). Voluntary and local government organizations were recruited as partners through which co-design, implementation, and data collection were carried out. To this end, stakeholder mapping was conducted at three levels: macro, meso and micro (1). Stakeholders were identified initially through the research team’s network of contacts which evolved iteratively through the mapping process to include more stakeholder relationships and included the following groups: (a) “Key Individuals” who held leadership roles within partner organizations or who were local health or voluntary sector experts operating through local health or government organizations, (b) “volunteers” who volunteered through one of partnered organizations, and (c) “community members” who lived in one of the research sites and who were potential clients of the research. Recruitment and sampling for the interviews and focus group was done through snowball sampling with initial study partner organizations identified during the stakeholder mapping process. Study partners assisted in the recruitment of the volunteers and community member focus groups and those sessions were held at the partner organization’s premises.

### Discussion Guide Development

Discussion guides were created for the different categories of respondents with questions and prompts adapted for the user needs. All discussion guides were semi-structured, had the same core structure and covered the same core questions. These included

a) How can the interventions be implemented?
b) What are the different stakeholders’ perspectives on the intervention?
c) What are the views and perceptions from the stakeholders on anticipated and current impact (what does impact mean for them in the field)?
d) What are the views and experiences on barriers and enablers of the intervention?
e) How can the interventions be sustained?

Development of the discussion guides was based on the Consolidated Framework for Implementation Research (CFIR) guidelines which have been used for a range of implementation applications (Keith et al., 2017). It a practical theory-based guide for systematically assessing potential barriers and facilitators to guide tailoring of implementation strategies and adaptations for the innovation being implemented and/or explain outcomes based on five overarching constructs.

### Data Collection

Face-to-face interviews or focus groups were carried out with study participants. All data from key individuals were collected through face-to-face interviews (*n* = 8) while all data from health volunteers and community members were collected from one of six focus groups (*n* = 39). Interviews were considered more appropriate for key individuals because of their specialist skills and experience and focus groups were considered more appropriate for community members and volunteers to gather a more diverse range of views and to encourage discourse. Data were collected in either the participant’s place of work or in community centers by an experienced anthropologist (P.N., PhD, female) and sessions were recorded and later transcribed verbatim for analysis by an external company (transcripts were not returned to participants before the analysis). No repeat interviews were conducted, but the researcher met some of the participants repeatedly before or after the interview in different community activities to maintain rapport. Interviews last for approximately 1 hr, and focus groups lasted for approximately 90 min. Participants were not reimbursed for their participation in this research.

### Data Analysis

Thematic analysis of qualitative data was carried out using a constant comparison method of analysis (Fram, 2013) which gathered and generated ideas and categorized them through an inductive process by three researchers using NVivo (IR [research fellow, PhD, female]/PN/ TGJ [research fellow, PhD, male). Line-by-line first-level coding of the transcripts was initially conducted on information pertaining to the research aims. These codes were then arranged into groups of meaningful concepts that made up the second-level codes. These secondary codes were reduced to the minimum number of themes that adequately captured all the data. The transcripts were then reviewed again by three researchers (IR/TGJ /CT [research assistant, BoM/BoS, female]) to explore dimensions of the themes and secondary codes thus refining the overall analysis. Finally, the research team used the themes and secondary codes to develop a series of recommendations.

## Results

### Stakeholder Mapping

At the macro level, stakeholders were identified through existing connections with the project’s principal investigator (PI) within the community. This was accomplished through meetings with local charity organizations, public health departments and other relevant voluntary sector organizations, as well as through online research. Interviews with these stakeholders yielded insights into sustainable implementation strategies. At the meso level, a strategic mapping exercise of community volunteer organizations was conducted to identify relevant organizations from which individual volunteers were selected. This process involved online searches, personal contacts, and snowball sampling.

Organizations and individuals were assessed to determine whether they met the inclusion criteria, such as a willingness to provide intervention services and engage in an iterative co-design process with community representatives (Boyd et al., 2012; Nahar et al., 2020). In-depth interviews were conducted to facilitate sustainable intervention implementation with these stakeholders.

At the micro level, mapping was undertaken with volunteers and end-user groups to co-design an acceptable and feasible intervention implementation. While the methodology involved co-designing the intervention with local stakeholders and volunteers, it is important to note that there was a skew toward female participants due to the higher representation of females in voluntary organizations. Furthermore, within the field area, there were a higher number of female service users than male service users utilizing the services of those organizations.

The stakeholder mapping exercises provided valuable context for recruitment and co-design of the intervention implementation and were instrumental in ensuring the relevance and feasibility of the study objectives (BSR, 2011; Nahar et al., 2020).

### Participant Characteristics

Participants were recruited through one of three partnerorganisations which were either voluntary sector organizations or local government organizations at the three research sites. Community members were also recruited in association with the partner organizations. A total of 47 individuals took part in this study. Data were collected on the amount of time that key individuals and volunteers had worked with the partner organizations. See Table 1 for participant characteristics.

**Table 1:** Participant Characteristics

	Key individuals			Volunteers			Community members			Total	
Organisation	n	Years^*^	m/f^+^	n	Y^**^	m/f^+^	n	Y^***^	m/f^+^	n	m/f
ORG 1	3	12	2/0	5	14	0/5	7	11.4	2/5	14	4/11
ORG 2	3	7	0/3	6	4	2/5	10	7	2/8	19	6/13
ORG 3	3	11.5	2/1	7	4.2	4/2	4	9.5	0/4	14	3/11
Total	8	10.8	4/4	18	8.25	5/13	21	9	4/17	47	13/34

* Average number of years working in the local community sector. **Average number of years spent associated with the organizations. ***average number of years spent living in the area. ^+^males/ females (based-upon self-identification). Note, key individuals were interviewed, and community members/volunteers took part in focus groups.

### Thematic Analysis

We identified three themes from the data which were (a) Management, (b) Logistics, and (c) Sociocultural factors.

### Theme 1—Management

This section focuses on the management of community partnerships, community health volunteers, and participants.

#### Meeting Community Needs

The SPICES project was felt to fit well with the current work and aims of the partner organizations, which included improving quality of life and delivering “partnership work” and a more “community-based offer.” It was also agreed that the project addressed a pressing health need in deprived communities:

*I think people should know more about their body than they do . . . There’s always a need.* (Community member ORG1 PAR2)

However, doubt was expressed over whether the need for a CVD intervention would be recognized by the community:

*Yeah it’s needed, but I don’t think the community thinks they’re needed [ . . . ] that’s the problem, no one will ever say I need it unless they’re literally in the back of an ambulance.* (Volunteer ORG2 PAR1)

The importance of avoiding “parachuting projects into communities” was also raised, with the avoidance of a “top-down approach” felt to be an advantage of SPICES:

*What I do see is that people generally want to see things done in a way that people feel more ownership of and to me that feels like we’ve got some chance of making this work[ . . . ] I’m getting the vibe from the process is that it’s trying to help local people understand some of the issues so[ . . . ] they can take some action and show some evidence of change to local people.* (Key Individual ORG1)

#### Participant Recruitment and Community Engagement

The idea of recruiting participants through a health and wellbeing event was repeatedly raised. It was felt an effective recruitment event should be fun and offer a range of activities in addition to screening, including children’s activities to attract families:

*If I went to someone down the street, do you want to go to a thing about heart disease, they would be like, why would I want to do that? There’s got to be a motive, there’s got to be something behind it to entice them out to do it.* (Community member ORG2 PAR2)

*What we often find with parents is[ . . . ] they will be drawn in by something for their children. [ . . . ] We use children as a hook with the parents.* (Key Individual ORG2)

*Provided it’s dressed up as a fun health and well-being day and not just a kind of clinical health and well-being day then I think we will get people involved.* (Volunteer ORG1 RES3)

Respondents also suggested advertising or recruiting for the project via General Practice (GP) surgeries, high street stalls, social media and community groups, and using personal or local stories to evoke an emotional connection to the project:

*There’s online, there’s events, there’s [ . . . ] groups that you can go and meet with [ . . . ] Tenants’ Associations or [ . . . ] local community groups and there’s things like newsletters and digital media.* (Key Individual ORG1)

Respondents anticipated that maintaining engagement with the project would be challenging, and that providing a program of ongoing support for participants would be key to this. Strategies suggested to maintain engagement included offering activity options to suit a range of demographics, offering child care if needed, and providing record cards so that participants could track their progress toward health goals:

*That would be the way to get the women involved, is that the kids can go alongside them, because they’ve got time, and especially if there’s like predesignated people to watch the children.* (Community member ORG1 PAR1)

*I’m saying give them like a records card or when every three months, this is where you was, this is how you are[ . . . ] the progress they’ve got in front of them so they can look at it, to give them something they can refer to it, otherwise they lose motivation very, very quickly.* (Volunteer ORG2 PAR3)

#### Volunteer Recruitment and Motivation

It was felt that to be most effective, volunteers should be embedded in the community they were working with, and ideally have experience of overcoming health problems and making lifestyle change themselves:

*I think one of the key things they probably need to be embedded in the community and talk the same language as the people they’re working with.* (Key Individual ORG2)

*The best people . . . to be able to talk about it are those who’ve had those experiences and how much they’ve benefitted from changing their life . . . because people are more likely to believe them and see if they can see the results.* (Volunteer ORG3 PAR7)

Conversely it was suggested that it would be difficult for an overweight volunteer to discuss heart disease risk with participants, and that there would be a need to “lead by example.”

*I think that I’m probably, you know, at least a stone and a half overweight [ . . . ] These conversations can be quite sensitive to have with people so it’s about, you know, how would I feel having these conversations and identifying other people’s risk when they might look at me and be like [laughs], do you know what I mean . . .* (ORG1 Volunteer PAR3)

Other characteristics of volunteers that were felt to be important were being committed, confident and outgoing.

Stated motivations for volunteering from volunteers included learning more about their local community, meeting new people, and “giving back” to the community or using and sharing skills:

*I’m a retired nurse and it was an opportunity to give something back, also we’re new to the area.* (Volunteer ORG3 PAR4)

*For me it was the outdoor activities [ . . . ] I just loved being outdoors.* (Community member ORG3 PAR5)

Improved job prospects and training were also suggested as possible motivations for volunteering, while time commitment associated with volunteering and travel were mentioned as potential barriers. It was felt that volunteer-led community groups would require a skilled co-ordinator, and that while these groups had the capacity to become self-sustaining, this would require ongoing periodic support from a partner organization.

#### Accessible and Supportive Communication

Respondents emphasized the need for “creating a bond” and engaging with people about their own circumstances and agenda, and tailoring communications according to an understanding of what each community might see as important. The importance of using motivational messages emphasizing the benefits of lifestyle change was repeatedly raised:

*Somehow if you could get across to people that this is for the benefit of them [ . . . ] Then you might get a better take up [ . . . ] it’s telling people the benefits they are going to get from doing it.* (Community member ORG2 PAR2)

It was agreed that simple words and messages should be used. Some respondents felt it was important to be “blunt” about lifestyle-related health risks, but that this should be balanced with positive messaging about the potential for change:

*. . .thinking about your idea of talking bluntly but at the same time being positive[ . . . ] if you’re in medium risk there would be symptoms you might be having[ . . . ] you know, would you like to be able to walk up the stairs without[ . . . ] losing breath[ . . . ] so it’s showing what the implications of being amber are, but putting it in a way that says you can change.* (Volunteer ORG1 PAR3)

The issue of low literacy was also raised, with concern that participants might find any written materials distributed as part of the intervention unclear or difficult to read:

*The average reading age in this country is nine or ten, and yet a lot of the literature we give out is really quite complicated [ . . . ] because a lot of them [ . . . ] may well have left school with minimal qualifications and even, their English or Maths might be fairly limited.* (*Key Individual ORG2)*

### Theme 2—Logistics

This section focuses on practical issues associated with the design and implementation of the intervention itself.

#### Structure of the Intervention

The relative merits of one-to-one versus group coaching sessions was discussed. It was felt that effective volunteer training would be required to successfully facilitate either type of session, but there was a general consensus that group session were preferable as they offered the potential to develop peer support networks:

*Actually it’s great! Because people find support from their peers [ . . . ] I think it’s also about the skills of the people who are facilitating that room to be able to address the dynamic in the group.* (Key Individual ORG3)

It was felt to be important that the program lasted long enough for participants to establish new healthy lifestyle habits, with ongoing support to deal with challenges and follow up key to success:

*It’s not about knowing[ . . . ] that’s the first stage[ . . . ] it only works when you have support, like if people could come back weekly or fortnightly or something and coming back[ . . . ] it’s to build a habit, and for building a habit it takes time, so you need to have people for at least six months coming back over and over and discussing the challenges that they had in their life in the meantime.* (Volunteer ORG3PAR)

*Well, if it’s going to carry on rather than have a set amount of time and then it all finishes [ . . . ] It’s follow up isn’t it?* (Community member ORG2 PAR4)

#### Using Technology

The pros and cons of utilizing technology and using an app-based tool to administer the screening questionnaires and support the intervention were discussed. Some respondents felt it would be useful to have an app showing how their heart disease risk reduced as they made lifestyle changes. It was also suggested that a WhatsApp group could be a good way to maintain peer support in-between intervention sessions:

*I was wondering about setting up[ . . . ] a communication like on WhatsApp[ . . . ]so people could be encouraged if they’re going through a little bit of a difficult time to communicate with the group[ . . . ] and sot of encouraging each other in between the meetings.* (Community member ORG2 PAR1)

However, some concerns were raised over levels of digital literacy, smart phone ownership and internet access in the community.

*There’s quite a lot of people won’t have a mobile phone or won’t have the skills to use it[ . . . ] our experience is people kept on changing their phone number because found out they’re in debt etc.[ . . . ] I think you need to think about both economic and skill levels.* (Key Individual ORG3)

*A lot of households don’t have internet. As much as it’s a common thing, a lot of households still don’t have it.* (Volunteer ORG1 PAR5)

#### Inclusion of Local Voluntary Services

The potential role for local voluntary services was raised several times. Benefits were felt to be both an increase in the range of activities/support that could be signposted to by the intervention, and the involvement of organizations that understood community needs:

*Community engagement is absolutely critical, because there isn’t enough capacity to be able to go to every individual [ . . . ] (and give them) motivational and personal trainer[ . . . ] so what you have to do is to try to get the maximum benefit from [ . . . ] an input which is shared with a number of people.* (Key Individual ORG3)

*With the GPs[ . . . ] they came[ . . . ] cause we desperately needed GPs and then they had a good look at the area[ . . . ] they got off their backsides and literally went out there and asked questions and talked to people[ . . . ] they came because they gave a damn about what had been going on in this community with previous doctors and they wanted to make a change.* (Community member ORG1 PAR2)

It was suggested local food organizations could be encouraged to participate and offer nutritional advice and support. Others suggested that healthy eating and cooking classes or gardening projects could be signposted to by SPICES-Sussex:

*So kind of an intergenerational gardening project could bring men that are experiencing loneliness [ . . . ] there’s something around how a garden can bring people together.* (Volunteer ORG3 PAR2)

#### Organizational Barriers and Facilitators to the Intervention

It was felt that volunteers would need training on heart disease to deliver the SPICES program, and that a program of activities to offer to participants would need to be put in place:

*The challenge isn’t doing the questionnaires [ . . . ] identifying the groups [ . . . ] the challenge is making sure we’ve got something to offer people.* (Volunteer ORG3 PAR3)

It was also suggested that community members might have difficulty accepting cardiovascular disease prevention as part of the partner organizations’ remit:

*Because people might think, well why are [they] doing stuff around cardiovascular disease when they’re a youth club.* (Volunteer ORG PAR1)

The difficulty of publicizing the intervention to individuals not already involved with partner organizations was also mentioned:

*I think a lot of it, you will find that everyone at [ORG1] has the same sort of group of people they can speak to so you’re not really spreading it, getting it out any further.* (Community member ORG1 PAR5)

Competing priorities, being “overloaded” and lack of funding were also raised as potential barriers to the intervention:

*People get overloaded and particularly professionals get overloaded. There’s so many events and so many things training to do.* (Key Individual ORG3)

However, it was generally felt that no real infrastructure changes would be required in the partner organizations, and that their established nature and contacts within communities would facilitate delivery of the program:

*[ORG3] has been here for 20 years now so in that time you get [ . . . ] you know a fair pool of contacts and connections just from being here.* (Volunteer ORG3 PAR1)

### Theme 3—Sociocultural Factors

This section focuses on the issues that relate to an individual’s psychological and mental health expectations and their previous life experiences.

#### Empowerment

Empowering individuals to gain a sense of control over their own health and thereby empowering the community to find their own tailored health solutions was felt to be crucial for the intervention to succeed:

*Cause if people think there’s a GP down the road that’s’ going to fix things for me all the time, or there’s a hospital that’s going to fix things for me all the time. . . so part of the barrier and the challenge is how do we get people to be more in control of their own health?* (Community member ORG1 PAR2)

Personal stories were said to be helpful to achieving this:

*“I think listening to people’s stories like your [focus group member’s name] is encouraging as well[. . .] just knowing that actually I felt the same way as you did and this is what happened to me[ . . . ] people like a story.”* Volunteer ORG2 PAR4.

It was suggested that communities selected for research or projects due to their social and health complexities could be disempowered if an adequate community consultation process was not undertaken:

*The other thing that this community’s had done to it lots and lots of times is that when local authorities and that come and do consultation [ . . . ] normally the decision’s already made and the consultation being done is just to back that up [ . . . ] and that’s again so disempowering to communities.* (Key Individual ORG1)

Utilizing existing successful community groups with an established culture of support was suggested as one way to promote empowerment through the intervention.

#### Community Context

Respondents highlighted the entrenched and intergenerational problems of poverty, lack of support structures, complex needs and ill-health faced by communities:

*You’ve got quite [ . . . ] high concentrations of really quite complex needs[ . . . ] there’s a big building site[ . . . ] about four hundred homes[ . . . ] they couldn’t sell them so they either sold or leased them to one of the big housing trusts who took a lot of complex families off the top of their housing list and dropped them into Peacehaven[ . . . ] without any support structures, either professional or personal.* (Key Individual ORG3)

These factors were felt to make engagement with local communities more difficult and to diminish motivation. Area-based stigma was also felt to be an issue:

*Generally this estate is considered to be quite a bad place. It’s always had that reputation ever since it was built in the 1930s and so what you have is quite a lot of [ . . . ] area-based stigma.* (Volunteer ORG1 PAR4)

Conversely, the strong volunteer basis, supportive nature, and potential for commitment if initial resistance could be overcome were noted as positive aspects of local communities:

*If you’ve got an issue everyone’s there for each other. Our kids, everyone knows our kids, so if something was to happen they all know, oh I’ll ring or I’ll do something. Everyone’s very close.* (Community member ORG1 PAR5)

#### Behavioral Barriers

Lack of time and other pressures were felt to be a major barrier to participation and to healthy lifestyle, particularly for parents, with child care, timing and location of intervention activities all being potential issues:

*I went to Zumba yesterday. That’s half five to half six, which is most people’s dinner, and bath time for their kids.* (Community member ORG1 PAR5)

Healthy eating was felt to present a number of challenges, including the cost and limited local availability of fresh fruit and vegetables, and the convenience of fast food compared to cooking:

*It is convenience isn’t it but a lot of parents if they can take them to McDonalds on the way home [ . . . ] yeah we can sit down and do something else for the rest of the evening.* (Community member ORG2 PAR4)

It was also felt that the chronic stress and pressures of coping day-to-day meant that for many, long-term health was not a priority:

*Your focus isn’t on what’s going to happen to me in five years’ time, the focus is just I need to get through today and then I need to get through tomorrow.* (Volunteer PAR4 ORG5)

*Their priority is you know, getting the kids to school on time, just having some food in the cupboard, so if you’re looking at getting through the day in that way you’re not going to be sitting down doing a food plan looking at how you meet the Government’s healthy eating guide.* (Key Individual ORG1)

#### Perceptions of Health

Denial of heart disease risk was felt to be a significant barrier to change, with the phrase “it won’t happen to me” being mentioned repeatedly. It was suggested that this might be partly because of the lack of obvious symptoms and invisible nature of heart disease:

*You can’t see if physically so [ . . . ] it’s like, we’re alright, we can get on with it. If you see something physically, like that’s a problem, we need to deal with it.* (Volunteer ORG1 PAR4)

*I think because you can’t see your heart can you, you can’t see what’s going on inside so you don’t know.* (Community member ORG1 PAR1)

It was also suggested that ill-health was normalized an unquestioned within the community, with healthy lifestyle being seen as something unachievable:

*People see people that go to the gym as being a million miles away of being anything that they can achieve.* (Key Individual ORG3)

#### Trust

The use of local trusted organizations was felt to be crucial to successfully delivering the intervention. Outside organizations were mistrusted within the communities due to broken promises and a lack of follow-up from previous community interventions:

*A lot of times people from the outside promise stuff and they never follow up on it, so to me that creates a barrier because people don’t trust them [ . . . ] they say we’ll give you all this, and they don’t get it.* (Community member ORG1 PAR1)

Developing a personal relationship with participants was considered important for effective health promotion, with community members being more receptive to taking health advice from local volunteers. It was also felt to be important that the volunteers trusted the organizations training them to deliver the intervention:

*By having people who are community messengers, who are people who are in the community, that trust, it stands a fighting chance of being heard.* (Key Individual ORG3)

It was suggested some participants might not trust the advice of volunteers who were not medically qualified:

*The trust element disappears a bit because you’re not in the doctor’s surgery. They’re then like, oh we can’t trust you, because you’re not a doctor.* (Volunteer ORG3 PAR5)

Conversely, it was felt that this lack of trust could be overcome if the volunteers seemed sufficiently knowledgeable, and it was pointed out that some community members mistrusted attending groups at the doctor’s surgery as they felt they were being watched:

*A lot of people don’t like going there ‘cause you’ve got social services haven’t you and the health visitors there, and a lot of people say, well, they’re watching.* (Community member ORG1 PAR5)

### Differences Between Respondent Groups

The three respondent groups present some differences in the way in they discussed CVD risk and the SPICES intervention. First, there were differences of opinion over communicating risk—volunteers and KIs felt that supportive communication and avoidance of “scary messages” were important, while community members felt that shocking messages might be needed to motivate people to change:

*. . . putting it harshly I think you might have to put the frighteners on people . . . I think actually say to them if you don’t live like this you will end up with this heat disease and that will cut ten years off your life.* (Community member ORG3 PAR3)

Second, in general community members tended to raise practical/logistical issues such as time, convenience, and child care as barriers to lifestyle change, whereas KIs were more likely to talk about issues like stress, deprivation, and competing priorities. They also discussed more macro level issues when discussing community involvement in the SPICES intervention. KIs often talked about issues of empowerment and bottom-up community development. This was not a major concern for community members and volunteers as they were more focused on pragmatic, and practical issues.

Finally, KIs and volunteers felt that lack of digital literacy could be a barrier to the use of technology in the intervention, whereas volunteers and community members were generally enthusiastic about technology and did not anticipate these problems:

*I think apps are really motivating [ . . . ] I work with people from 28 all the way up to some of them are late 60s with the changes in employment law, and I think you know, we make assumptions about older people not being very good with technology, but I think quite a lot of my older clients do actually use the technology as well.* (Community member, ORG2 PAR1)

## Discussion

This study had three objectives which facilitated the broader goal of developing a community-led CVD risk-assessment and coaching intervention as part of the pre-implementation phase of the SPICES-Sussex project (Nahar et al., 2020). First, it helped to identify and establish partnerships with local community organizations through which the main intervention could be delivered (objective 1). The analysis offered insights which helped to develop understanding of community and volunteer views and perspectives on the intervention (objective 2). Finally, the analysis of stakeholder interviews has allowed the research team to develop key recommendations for the design and delivery of the intervention (Objective 3). These recommendations will be presented later in the discussion section. Having completed the pre-implementation phase of the project, the team used this evidence as the starting point for the intervention development and delivery in the per-implementation phase of the project using co-design and community-based implementation approaches. This work will be presented in a later paper.

### Implications for Practice

This work supports concepts of community outreach and collaborative care in marginalized communities by a showing an openness to a community engagement-based model of CVD prevention (Gaziano et al., 2015; Usher-Smith et al., 2017). Chen *et al.* have argued there is a mismatch between populations in which interventions are validated and the populations in which they will be used (Chen et al., 2013). Meaningfully engaging with communities and acting on their views, experience, and guidance during the design of interventions can lead to more effective interventions during implementation. This tailoring is especially important when designing interventions for seldom heard, vulnerable populations and/or groups with differing socio-cultural backgrounds which are not well-represented in the workforce of researchers, policy makers, and intervention designers (Berra et al., 2017). Participants expressed a desire to engage in co-design of the project as previously observed by researchers (Donetto et al., 2015; O’Brien et al., 2011). Our results also emphasize the importance of the role of trust to the success of all aspects of the intervention, from willingness to engage in the co-design process, to CHV training and participant willingness to accept health coaching, and the potential for mistrust of external organizations who mishandle the process of community engagement (Jagosh et al., 2015).

A second key implication for intervention design was the importance of sociocultural factors in developing interventions; particularly empowering people to take an active role in designing health policies and interventions (Mulvale et al., 2019). This may go some way to addressing issues of low self-efficacy of health in marginalized communities (Rose & Hatzenbuehler, 2008; Wallerstein, 2002) by promoting participation, shared ownership and social capital (Edwards Jr, 2019; Palmer et al., 2019; Wildman et al., 2019). Service providers should also consider the practical and economic barriers to attending sessions and adopting healthy behaviors faced by members of marginalized communities.

### Recommendations

We developed the outcomes of the thematic analysis into a set of recommendations which may be helpful to other researchers and service providers.

1. To adopt a bottom-up approach to intervention design and evaluation through co-design.
2. To run interventions in close partnership with established community organizations with knowledge of the local area.
3. To focus on recruiting and retaining skilled and emotionally engaged volunteers who are embedded in the local community and who have their own experiences and stories regarding health.
4. To use group sessions to build peer support networks among intervention participants. Consequently, volunteers should be trained in managing group dynamics.
5. To focus on enjoyment of the sessions, and emotional bonds between participants.
6. To keep messaging and communication simple, clear and understandable and consider different ages, physical abilities and other circumstances.
7. To consider a range of offers and incentives they can make to CHVs and participants.

### Strengths and Limitations

Sampling for this study followed the stakeholder mapping process, and all participants were recruited based on links with the community partnership organization and/or the research team. This approach means that we may have lost some community voices because they lacked the links with the community partners. For example, this approach meant that the interviewees and focus group members were highly skewed toward older, female participants. This is due to the realist approach taken during this research as we have prioritized pathways and mechanism through which the intervention can be successfully designed and developed over issues of representativeness and Generalizability (Pawson, 2006). We believe our approach to sampling was justified as future scaling up of the project is to be carried out through the participating community organizations. The stakeholder mapping stage of the research highlighted several community organizations through which the intervention could be implemented, and those partners largely worked with a female volunteer base. Furthermore, is it known that women are more likely to fill voluntary roles in the United Kingdom with care and support roles being especially skewed toward women (Department for Digital, Culture, Media & Sport, 2021; UN Volunteers, 2022). In addition, the findings may not be generalisable to other contexts given the focus on local issues during the focus groups and interviews. We argue that confidence in the transferability of the headline findings is enhanced by the internal consistency in the findings from stakeholders at different sites who are involved at different levels of the future intervention, and consistency with previous literature. In addition, interviews were carried out before the Covid-19 pandemic. Community organizations may have substantially shifted their ways of working during the pandemic; however, we feel our recommendations are still relevant and appropriate even in the pandemic context and beyond.

## Conclusion

This study showed an openness to the SPICES CHV-led intervention model for preventing CVD. The community members displayed willingness to engage in the collaborative co-design design of the SPICES-Sussex project, and highlighted issues of empowerment, ownership, and sustainability as important for their participation.
